# Chronic obstructive pulmonary disease (COPD) and COPD-like phenotypes

**DOI:** 10.3389/fmed.2024.1375457

**Published:** 2024-04-09

**Authors:** Spyridon Fortis, Dimitris Georgopoulos, Nikolaos Tzanakis, Frank Sciurba, Joseph Zabner, Alejandro P. Comellas

**Affiliations:** ^1^Center for Access and Delivery Research and Evaluation, Iowa City VA Health Care System, Iowa City, IA, United States; ^2^Division of Pulmonary, Critical Care and Occupational Medicine, Department of Internal Medicine, University of Iowa, Iowa City, IA, United States; ^3^Medical School, University of Crete, Heraklion, Greece; ^4^Division of Pulmonary, Allergy and Critical Care Medicine, Department of Medicine, University of Pittsburgh, Pittsburgh, PA, United States

**Keywords:** chronic obstructive pulmonary disease, phenotypes text word count: 2, chronic hypercapnic respiratory failure, chronic hypoxemic respiratory failure, preserved ratio impaired spirometry (PRISm), hyperinflation, preserved spirometry at risk for COPD, frequent respiratory exacerbations

## Abstract

Chronic obstructive pulmonary disease (COPD) is a heterogeneous disease. Historically, two COPD phenotypes have been described: chronic bronchitis and emphysema. Although these phenotypes may provide additional characterization of the pathophysiology of the disease, they are not extensive enough to reflect the heterogeneity of COPD and do not provide granular categorization that indicates specific treatment, perhaps with the exception of adding inhaled glucocorticoids (ICS) in patients with chronic bronchitis. In this review, we describe COPD phenotypes that provide prognostication and/or indicate specific treatment. We also describe COPD-like phenotypes that do not necessarily meet the current diagnostic criteria for COPD but provide additional prognostication and may be the targets for future clinical trials.

## Introduction

**FIGURE 1:**
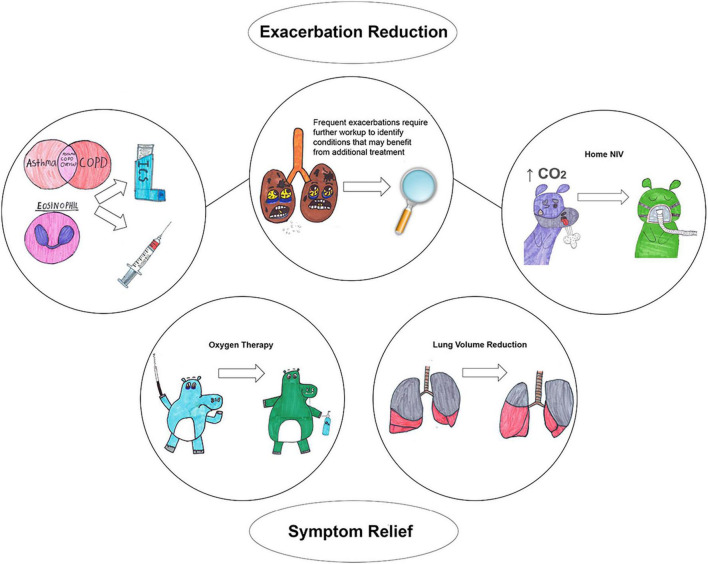
Clinically relevant COPD phenotypes and corresponding treatments.

Chronic obstructive pulmonary disease (COPD) is defined as persistent respiratory symptoms and airflow limitation due to airway and/or alveolar abnormalities usually caused by noxious particles or gases and influenced by host factors (1). COPD diagnosis requires not only a consistent history but also the presence of airflow limitation via spirometry. Historically, two phenotypes have been described to further characterize the disease: *chronic bronchitis*, which is associated with airway inflammation with the main characteristic being chronic productive cough, and *emphysema*, which is associated with alveolar destruction and presents with dyspnea. Although these phenotypes provide some additional characterization of the pathophysiology of the disease, they are not extensive enough to reflect the heterogeneity of COPD and do not provide granular categorization that indicates specific treatment, perhaps with the exception of adding inhaled glucocorticoids (ICS) in patients with chronic bronchitis (2). Moreover, the current definition of COPD does not include individuals with high respiratory burden, e.g., high mortality and frequent respiratory hospitalizations, that do not have airflow limitation (3–5). The first objective of this review is to describe clinically relevant COPD phenotypes that provide prognostication and/or indicate specific treatment (Figure 1). The second objective is to describe COPD-like phenotypes that do not necessary meet the current diagnostic criteria for COPD but provide additional prognostication and may indicate response to certain treatments that can be tested in future clinical trials. Table 1 provides a summary of all phenotypes.

**TABLE 1:** Summary of chronic obstructive pulmonary disease (COPD) and COPD-like phenotypes.

Phenotypes	
Asthma-COPD overlap and COPD with eosinophilia	High eosinophils in patients with COPD indicate obstructive lung disease that responds to ICS, and high eosinophils and BDR may indicate disease that responds to biological therapy.
COPD with static hyperinflation	Lung volume reduction in selected patients with severe lung function impairment and static hyperinflation is associated with improvement in lung function, exercise capacity, and quality of life.
COPD with chronic hypoxemic respiratory failure	Oxygen supplementation in patients with COPD and resting chronic hypoxemic respiratory failure improves survival but is not beneficial in those with isolated exertional or nocturnal hypoxemia.
COPD with chronic hypercapnic respiratory failure	Home nocturnal non-invasive ventilation in patients with COPD and chronic hypercapnic respiratory failure improves survival and reduces hospitalizations.
COPD with frequent respiratory exacerbations	Frequent respiratory exacerbations may indicate escalation of treatment (ICS, azithromycin, roflumilast, home NIV) and further investigation is required to identify other conditions, e.g., antibody deficiency syndrome, that may benefit from additional treatment.
Preserved spirometry at risk for COPD	Individuals with normal spirometry and history of smoking who have chronic bronchitis or respiratory exacerbations have increased respiratory-related hospitalizations and mortality. Further research is needed to assess preventive treatment for individuals with normal spirometry but who are at risk of progressing to COPD.
Preserved ratio impaired spirometry (PRISm)	PRISm is a volatile spirometric pattern associated with respiratory symptoms and mortality. Further research is needed to confirm whether individuals with PRISm benefit from existing treatment for COPD.

## Asthma-COPD overlap and COPD with eosinophilia

Asthma-COPD overlap is a relatively new term that describes the coexistence of asthma and COPD (6). Definitions have been proposed by scientific groups including the Global Initiative for Chronic Obstructive Lung Disease (GOLD) and the Global Initiative for Asthma (GINA) but none offer prognostication and do not indicate specific treatment (7). Because bronchodilator responsiveness (BDR) is one of several criteria to confirm lung function variability, (8) BDR presence is often and erroneously considered equivalent to asthma diagnosis. Although BDR is more common and greater in patients with asthma than those with COPD, BDR cannot distinguish asthma and COPD (9–11). Further, the association of BDR with clinical outcomes in patients with COPD has been extensively studied with conflicting findings (12–14). Early studies showed that BDR cannot predict response to treatment (12–14). The only outcome which is consistently associated with BDR is FEV_1_ decline over time (15–17). The inconsistent findings regarding the association of BDR and clinical outcomes may be related to various BDR definitions and protocols. In COPD, an increase in both FEV_1_ and FVC by 12% and 200 ml after bronchodilator administration was associated with a phenotype that is characterized by less emphysema but reduced mortality (15). BDR in FVC is typically associated with greater degree of emphysema (18, 19) and functional small airway disease (20). A drawback of BDR is its instability over time (14, 15). Nevertheless, the hallmark of asthma is lung variability, and it is not surprising that BDR is not stable over time. A recent study showed that consistent BDR in individuals with former or current smoking exposure is associated with prior asthma diagnosis, lung function decline, and functional small airway disease (20).

Eosinophils are strongly tied with Th2-mediated inflammation, historically considered the type of inflammation that occurs in asthma, as opposed to Th1-mediated inflammation, typically present in COPD (6). Increased sputum and blood eosinophil counts indicate response to ICS and patients with COPD who have blood eosinophil counts ≥ 300 cells/μL should consider adding ICS combined with bronchodilator treatment (Figure 1) (1). Biologic agents that target interleukin-5, interleukin-5 receptor and interleukin-4/interleukin-13 receptor in patients with COPD and elevated blood eosinophil counts reduce exacerbations (Figure 1) (21, 22). A post-hoc analysis of two randomized placebo controlled trials in COPD determined that the anti-IL5 agent benralizumab had greater response in patients with BDR in FEV_1_ (23). This is the first study showing that BDR can indicate response to a specific pharmacological agent.

*Summary: High eosinophils in patients with COPD indicate obstructive lung disease that responds to ICS and biologics, and BDR may indicate disease with greater response to biological therapy.*

## COPD with static hyperinflation

Hyperinflation often occurs in COPD as the disease progresses and is well known to be associated with poor prognosis (24–27). The landmark study by Casanova et al. (25) showed that hyperinflation, defined as decreased inspiratory capacity to total lung capacity (TLC) ratio, is associated with increased mortality. More recently, studies confirm those findings using other definitions of hyperinflation (26, 27). Several definitions exist which refer to whether measurements of hyperinflation occur during rest (static) or exercise (dynamic) and the measurement themselves, e.g., functional residual capacity, residual volume (RV) (28).

Lung volume reduction surgery can alleviate static and dynamic hyperinflation and improve outcomes, including mortality (Figure 1) (29, 30). In one study, patients with upper-predominant emphysema, low post-rehabilitation exercise capacity who underwent lung volume reduction surgery had a long-term survival benefit (29). Nevertheless, lung volume reduction surgery is associated with high perioperative mortality, limiting the enthusiasm for the procedure (31). Recently, endobronchial valves placed bronchoscopically, a significantly less invasive procedure than lung resection, have been used as an alternative method to reduce hyperinflation. Endobronchial valves decrease the size of hyperinflated areas of the lung by allowing air to exit the hyperinflated areas but not to re-enter it. By reducing hyperinflation, endobronchial valves have been shown to improve lung function, exercise capacity, quality of life, and may reduce mortality (24, 32, 33). Patients with COPD should not only have severe lung function impairment (FEV1% predicted < 45%) and static hyperinflation (TLC > 100% and RV > 175%) but also need to have significantly reduced exercise capacity (6-min walk distance < 450 m or 1,476 feet) to benefit from endobronchial valves (34). Moreover, these patients should not have severe alveolar gas exchange impairment (PaO_2_ < 45 mmHg, and PaCO_2_ > 50 mmHg) and significantly impaired exercised capacity which can increase the periprocedural risk. Chronic bronchitis is one of the exclusion criteria as it can be associated with difficultly clearing airways of the treated areas and risk of respiratory infections. Insertion of endobronchial valves are associated with 25–30% risk for pneumothorax and for that reason requires inpatient observation for 3 days (32).

*Summary: Lung volume reduction in selected patients with severe lung function impairment and static hyperinflation is associated with improvement in lung function, exercise capacity, and quality of life.*

## COPD with chronic hypoxemic respiratory failure

In the advanced stages of COPD, hypoxemia, defined as oxygen saturation below 89%, may develop. Oxygen supplementation in patients with COPD and chronic hypoxemic respiratory failure at rest has shown remarkable long-term mortality benefit with more than 50% increase in survival (Figure 1) (35, 36). However, the benefit of oxygen supplementation in isolated hypoxemia on exertion (absence of hypoxemia at rest) is either minimal or absent (37–39). Emtner et al. (38) showed that oxygen supplementation increased exercise capacity in patients undergoing exercise training. Patients who received oxygen supplementation while exercising were able to train 4 min longer than those who did not receive oxygen. Nonoyama et al. (37) showed that oxygen supplementation improved a 5-min walk test by just 15 steps relative to air supplementation (control) (37). A large meta-analysis showed that oxygen supplementation in patients with COPD and isolated exertional hypoxemia does not improve any clinical outcome (40). Supplemental oxygen in those with moderate hypoxemia is also not beneficial (39, 40). Oxygen supplementation in those with isolated nocturnal hypoxemia has no benefit despite that these patients may be hypoxemic more than half of the duration of their sleep (41, 42).

*Summary: Oxygen supplementation in patients with COPD and resting chronic hypoxemic respiratory failure improves survival but it is not beneficial in those with isolated exertional or nocturnal hypoxemia.*

## COPD with chronic hypercapnic respiratory failure

Chronic hypercapnic respiratory failure is another consequence of COPD that occurs in advanced stages of the disease and is associated with increased mortality (43–45). The pathophysiological mechanism of chronic hypercapnic respiratory failure is complex and not entirely understood but hypoventilation seems to play the primary role (46, 47). During sleep, hypoventilation is more pronounced and nocturnal home non-invasive ventilation (NIV) has been used in these patients (48). Early studies of home nocturnal NIV in those patients showed no benefit (49, 50) but recent randomized controlled trials (RCTs) showed improvement in clinical outcomes, including reduction in hospitalizations and mortality benefit (Figure 1) (51, 52). Recent meta-analyses found an improvement in mortality, hospitalizations, dyspnea, exercise capacity, and health-related quality of life relative to standard treatment (53, 54). It seems that the benefit is related to a particular ventilator strategy, known as high intensity, that includes large inspiratory to expiratory airway pressure difference, high minute ventilation, and reducing baseline CO_2_ levels by 25%, as well as with selection of patients with severe disease, those with FEV_1_% predicted < 50% and PaCO_2_ > 52 mmHg (51, 52, 55). RCTs with favorable outcomes recruited patients with severe lung function impairment and hypercapnia who had a recent COPD-related hospitalization or chronic hypoxemic respiratory failure (51, 52, 56). A recent metanalysis showed that both higher baseline arterial CO_2_ levels and greater magnitude of the CO_2_ reduction from NIV are associated with greater improvement in clinical outcomes (53). Nevertheless, this may merely reflect that these patients are sicker and benefit more from treatment. Despite the significant benefits from home nocturnal NIV, it is underutilized among those with COPD-related hospitalizations, with less than 3% of those patients using bilevel positive airway pressure/home NIV (57, 58).

*Summary: Home nocturnal non-invasive ventilation in patients with COPD and chronic hypercapnic respiratory failure improves survival and reduces hospitalizations.*

## COPD with frequent respiratory exacerbations

This phenotype of COPD has attracted a lot of attention despite the small proportion of patients with this phenotype, due to fact that this group of patients consume the largest proportion of healthcare resources and have poor prognosis (59, 60). Beeh et al. (59), in a sample of patients with moderate or severe COPD, showed that 14% of the patients accounted for 57% of the total COPD-related hospitalizations. In our previous work, among patients with COPD and mild-to-moderate lung function impairment, the top 5% in exacerbation frequency accounted for 34% of the total exacerbations in the cohort (60). Those with frequent exacerbations have increased mortality relative to those with no exacerbations (60). Although a formal definition for this phenotype does not exist, at least two moderate exacerbations or one hospitalization has been used as a cutoff to identify patients with COPD who require escalation of treatment according to GOLD guidelines (1). Patients with frequent exacerbations may benefit from addition of ICS on bronchodilator therapy, azithromycin, and roflumilast.

This phenotype is heterogenous and likely includes patients with chronic bronchitis, asthma-COPD overlap, and chronic hypercapnic respiratory failure (Figure 1). Thus, patients with frequent exacerbations and asthma-COPD overlap may benefit from the addition of ICS and/or biological therapy (1, 21, 23). Patients with frequent exacerbations and chronic hypercapnic respiratory failure may see a reduction in hospitalizations with home NIV use (61). Another comorbidity should be considered with this phenotype, e.g., antibody deficiency syndrome, which may increase respiratory exacerbations (62, 63). A retrospective study of patients with COPD and antibody deficiency syndrome with frequent exacerbations showed that appropriate treatment including cycling antibiotics or IgG supplementations reduced respiratory exacerbations from median of four to one exacerbation every year (64).

*Summary: Frequent respiratory exacerbations may indicate escalation of treatment (ICS, biologics, azithromycin, roflumilast, home NIV) and further investigation is required to identify other conditions*, e.g., antibody deficiency syndrome, *that may benefit from additional treatment.*

## Preserved spirometry at risk for COPD

Recently, this phenotype has been the focus of several investigations because it may reflect a state or condition that precedes COPD and may benefit from early treatment. Woodruff et al. (4) showed that among individuals with at least 20-pack year of current or former smoking exposure, CAT score > 10 (highly symptomatic) was associated with increased respiratory exacerbations and hospitalizations (4). Pooling individual data of 5 prospective cohorts, Balte et al. (65) showed that non-obstructive chronic bronchitis (chronic bronchitis with normal spirometry) was associated with respiratory-related hospitalizations and mortality (65). A metanalysis confirmed that non-obstructive chronic bronchitis was associated with increased all-cause mortality in individuals with current or former smoking exposure but not in people without history of smoking exposure (5). Air trapping in individuals with current or former smoking exposure is associated with increased medication use, respiratory hospitalizations, progression to COPD, and mortality (5, 66). Regan et al. (67) showed that among people with at least 10 pack-years of current or former smoking exposure, 42% have features consistent with obstructive lung disease in the chest CT (67). Respiratory exacerbations in individuals with normal lung function and current or former smoking exposure are associated with lung function decline and increased all-cause mortality (60). It is possible that progression to COPD is mediated by lung function decline that results from respiratory exacerbations in individuals with normal lung function. COPDGene investigators have led an effort to expand the definition of COPD to include individuals without spirometric obstruction who are at risk for lung function decline or death (3).

A recent RCT showed that dual bronchodilator treatment in individuals with smoking history and respiratory symptoms but relative normal spirometry defined as post-bronchodilator FEV_1_/FVC ≥ 0.7 and FVC % predicted ≥ 70% did not improve respiratory symptoms (68). However, the lack of efficacy could be due to the fact that bronchodilators have minimal effect on individuals with near normal lung function. It is possible that other types of pharmacotherapy in a different group of individuals should be tested, e.g., inhaled corticosteroids in individuals with non-obstructive chronic bronchitis or respiratory exacerbations. Identifying individuals with normal lung function at risk for COPD and lung function decline may be the focus of future therapeutic trials that will test pharmacological agents which can prevent progression to COPD.

*Summary: Individuals with normal spirometry and history of smoking who have chronic bronchitis or respiratory exacerbations have increased respiratory-related hospitalizations and mortality. Further research is needed to assess preventive treatment of individuals with normal spirometry but who are at risk of progressing to COPD.*

## Preserved ratio impaired spirometry (PRISm)

Preserved ratio impaired spirometry (PRISm), also known as restrictive or unclassified spirometry, is a common spirometric pattern which occurs in 10–20% of spirometries (69–72). PRISm is usually defined as reduced FEV_1_ with a normal FEV_1_/FVC (69) but other definitions applied also refer to a non-obstructive abnormal spirometry (70). General population studies have reported that it is associated with increased all-cause mortality (70, 73). Studies in individuals with current or former smoking exposure have shown that PRISm is a heterogenous group with significant symptoms and reduced exercise capacity that includes patients with an FEV_1_% predicted that can range from 44 to 79%, a body mass index (BMI) between 17.2 and 53.8 kg/m^2^, and radiographic emphysema on chest CT that can range from < 1% to up to 11% (69). Individuals with PRISm have higher BMI relative to patients with normal spirometry or obstruction and it has been postulated that PRISm merely is the result of high BMI. However, a landmark study by Jones and Nzekwu (74) showed that although BMI is inversely associated with FVC and total lung capacity (TLC), obesity in individuals with no respiratory disease is unlikely to reduce FVC below the lower limit of normal (LLN). One study demonstrated that among patients undergoing preoperative evaluation for bariatric surgery with a BMI of ≥ 35 Kg/m^2^, only 3% had an FVC < LLN (75). Although a proportion of PRISm could be related only to obesity, it is unlikely that obesity is the sole cause of PRISm in the majority of the cases. Other diseases such as interstitial lung diseases (ILD) can cause PRISm (72) but the prevalence of ILD is extremely low and thus it is unlikely that ILD accounts for the majority of PRISm cases. Cohorts such as COPDGene, with a PRISm prevalence of 12–15%, exclude ILDs (69, 76).

Most individuals with PRISm likely have obstructive lung disease. Approximately half of individuals with PRISm have TLC on chest CT and < 80% predicted or LLN, which likely results from obesity (69). Obesity is associated with increased FEV_1_/FVC in patients with COPD (77). Lower TLC secondary to obesity may result in pseudo-normalization of FEV_1_/FVC ratio and underdiagnosis of obstructive lung disease. Our previous work has shown that air trapping in PRISm is associated with increased respiratory exacerbations, progression to COPD, and increased mortality (78). Unfortunately, no clinical trials have assessed the effect of existing pharmacotherapies on PRISm. Conducting clinical trials in PRISm is a difficult task as PRISm is an unstable phenotype (76, 79). It has been reported that 25% of individuals with PRISm have COPD on future spirometries, and 16% of those with PRISm had COPD in prior spirometries (76).

*Summary: PRISm is a unstable spirometric pattern associated with respiratory symptoms and mortality. Further research is needed to confirm whether individuals with PRISm benefit from existing treatment for COPD.*

## Conclusion

More granular phenotyping of COPD (asthma-COPD overlap, hyperinflation, chronic hypercapnic respiratory failure, frequent respiratory exacerbations) can help to identify patients that respond well to existing treatments, e.g., ICS, home nocturnal NIV. Further research is needed in those individuals at risk for COPD who do not yet have obstructive spirometry (normal spirometry or PRISm) to assess whether treatment can improve outcomes including preventing progression to COPD.

## Author contributions

SF: Conceptualization, Writing – original draft, Writing – review & editing. DG: Supervision, Writing – review & editing. NT: Supervision, Writing – review & editing. FS: Writing – review & editing. JZ: Supervision, Writing – review & editing. AC: Supervision, Writing – review & editing.
